# Testing *S. sonnei* GMMA with and without Aluminium Salt-Based Adjuvants in Animal Models

**DOI:** 10.3390/pharmaceutics16040568

**Published:** 2024-04-22

**Authors:** Francesca Mancini, Valentina Caradonna, Renzo Alfini, Maria Grazia Aruta, Claudia Giorgina Vitali, Gianmarco Gasperini, Diego Piccioli, Francesco Berlanda Scorza, Omar Rossi, Francesca Micoli

**Affiliations:** 1 GSK Vaccines Institute for Global Health S.r.l. (GVGH), 53100 Siena, Italy; 2 Dipartimento di Biotecnologie Mediche, Università degli Studi di Siena, 53100 Siena, Italy; 3 GSK, 53100 Siena, Italy; claudia.g.vitali@gsk.com (C.G.V.);

**Keywords:** adjuvant, OMV, GMMA-based vaccine, unadsorbed GMMA, immune response

## Abstract

Shigellosis is one of the leading causes of diarrheal disease in low- and middle-income countries, particularly in young children, and is more often associated with antimicrobial resistance. Therefore, a preventive vaccine against shigellosis is an urgent medical need. We have proposed Generalised Modules for Membrane Antigens (GMMA) as an innovative delivery system for *Shigella sonnei* O-antigen, and an Alhydrogel formulation (1790GAHB) has been extensively tested in preclinical and clinical studies. Alhydrogel has been used as an adsorbent agent with the main purpose of reducing potential GMMA systemic reactogenicity. However, the immunogenicity and systemic reactogenicity of this GMMA-based vaccine formulated with or without Alhydrogel have never been compared. In this work, we investigated the potential adjuvant effect of aluminium salt-based adjuvants (Alhydrogel and AS37) on *S. sonnei* GMMA immunogenicity in mice and rabbits, and we found that *S. sonnei* GMMA alone resulted to be strongly immunogenic. The addition of neither Alhydrogel nor AS37 improved the magnitude or the functionality of vaccine-elicited antibodies. Interestingly, rabbits injected with either *S. sonnei* GMMA adsorbed on Alhydrogel or *S. sonnei* GMMA alone showed a limited and transient body temperature increase, returning to baseline values within 24 h after each vaccination. Overall, immunisation with unadsorbed GMMA did not raise any concern for animal health. We believe that these data support the clinical testing of GMMA formulated without Alhydrogel, which would allow for further simplification of GMMA-based vaccine manufacturing.

## 1. Introduction

*Shigella* infections are one of the top causes of moderate-to-severe diarrheal disease in developing countries and among children younger than 5 years, and no vaccine is currently widely available against this pathogen [1,2,3,4]. The O-antigen (OAg) component of the lipopolysaccharides (LPS) has been recognised as a key protective antigen [5,6,7,8], and *Shigella* OAg-based vaccines are currently under clinical development [9,10,11,12].

We have proposed Generalised Modules for Membrane Antigens (GMMA) as an innovative delivery system for *S. sonnei* OAg with the intent to develop a vaccine to prevent *S. sonnei* infections, as the basis for a broader four-component protective vaccine [13]. GMMA are outer membrane vesicles (OMVs) from Gram-negative bacteria genetically engineered to enhance their release [13] and reduce the reactogenicity after injection by modifying the lipid A structure, still maintaining the lipid A-triggered immunopotentiator effect of the Toll-like receptor (TLR)4 [14]. A rapid and simple manufacturing process allows high yields of GMMA to be obtained, with the potential for low-cost vaccines [15]. Alhydrogel has been used for *S. sonnei* GMMA vaccine formulation as an adsorbent agent with the main purpose of reducing potential GMMA systemic reactogenicity [16,17,18]. Indeed, besides triggering innate immune cell responses, it has been proposed that an aluminium salt-based adjuvant ensures the formation of a depot at the injection site, which would reduce the systemic exposure of immunogens adsorbed on it [19,20]. The addition of an adjuvant to achieve protective immunity is usually required for formulations made from pure antigens that tend to have low immunogenicity [21]. By contrast, GMMA contain pathogen-associated molecular patterns (PAMPs) of the pathogen they originate from, e.g., TLR4- and TLR2-activating components, and therefore per se possess the ability to stimulate the innate compartment of the immune system without the need of an adjuvant [22]. GMMA not only possess TLR-activating components that can confer self-adjuvanticity [14,22].

In this work, we evaluated the immunogenicity of *S. sonnei* GMMA in different animal models when administered alone or in combination with aluminium salt-based adjuvants, i.e., Alhydrogel or AS37. AS37 is an Adjuvant System containing a synthetic TLR7 agonist adsorbed onto aluminium hydroxide [23]. We tested AS37 since it could complement the TLR4 and TLR2 activation properties already possessed by GMMA. Indeed, TLR7 and TLR4 agonists have been previously shown to induce synergistic increases in antigen-specific responses [24,25,26,27]. Here, we performed an in-depth investigation on the impact that aluminium salt-based adjuvants can have on GMMA immunogenicity, and we gained a first insight into the tolerability of GMMA formulated without Alhydrogel in rabbits.

## 2. Materials and Methods

### 2.1. Bacterial Strains and Generation of Mutants

The *S. sonnei* strain was received by WRAIR and modified as previously described in [13]. Briefly, the virulence plasmid-encoded *virG* gene was replaced with the *nadA* and *nadB* genes from *E. coli*. The *tolR* gene was replaced with the kanamycin resistance gene *aph*. The *msbB* genes were replaced with the erythromycin and chloramphenicol resistance genes *erm* and *cat*.

### 2.2. GMMA Production and Characterisation

Shigella GMMA were produced and purified from the *S. sonnei* 53G Δ*tolR::kan* Δ*virG::nadAB* Δ*msbB2::cat* Δ*msbB::erm* strain, as previously described [13].

The total protein content was estimated by microBCA using bovine serum albumin (BSA) as a reference following the manufacturer’s instructions (Thermo Scientific, Waltham, MA, USA), while the total OAg amount was determined by high-performance anion-exchange chromatography with pulsed amperometric detection (HPAEC-PAD) [28]. OAg molecular size was determined by size-exclusion high-performance liquid chromatography (HPLC-SEC) after acetic acid extraction [28]. GMMA particle size was determined by dynamic light scattering, soluble proteins were measured by microBCA after GMMA separation by ultracentrifugation, and lipid A content was estimated by reversed-phase high-performance liquid chromatography (HPLC-RP MS)/HPAEC-PAD [28].

### 2.3. GMMA Formulation

GMMA tested without Alum-based salts were diluted with saline at the different concentrations tested in the animal studies. For the formulations with Alhydrogel, GMMA were diluted with water to reach a final concentration of 26.7 µg OAg/mL. Alhydrogel was added to reach a concentration of 0.7 mg Al3+/mL in the final formulated bulk, following the process previously described [13]. This bulk formulation was diluted with the Alhydrogel diluent (0.7 mg Al3+/mL Alhydrogel) to reach the OAg concentrations tested in the animal studies.

For the formulations with AS37, GMMA were diluted at the different concentrations tested in the animal studies directly. Alhydrogel and LHD153 were mixed in water, and then 100 mM histidine buffer at pH 6.5 was added to reach a 10 mM concentration in the final formulation. Sodium chloride (1540 mM) was then added, yielding 154 mM in the final formulation. Finally, GMMA diluted in saline were added. Formulations were gently mixed by inversion. The LHD153 dose for this study was selected based on previous work by Buonsanti et al. [29]. All formulations were stored at 4 °C until immunisation.

GMMA formulations were tested to verify the complete adsorption of GMMA on Alhydrogel via silver staining, by comparing the formulation supernatant after Alhydrogel removal by centrifugation with GMMA at known concentrations. LHD153 adsorption on Alhydrogel was verified by running the supernatants on HPLC-RP (Acquity BEH C18 column with a gradient of 0.1% TFA in water and 0.1% TFA in acetonitrile) in comparison to a standard curve built with LHD153 in the range of 3.0–48.8 µg/mL.

### 2.4. Animal Studies

All animal studies were performed at the GSK Animal Resources Centre under the animal project 471/2020-PR approved by the Italian Ministry of Health. Groups of 8 CD1 mice (female, 5 weeks old) were vaccinated intramuscularly with 36–3.6–0.36–0.036 ng of GMMA (OAg dose based on HPAEC-PAD quantification). Briefly, 50 µL of the vaccine was administered to each mouse on study days 0 and 28 either in the absence or presence of 0.7 mg/mL of Alhydrogel (Al3+) or AS37 (2 mg/mL aluminium hydroxide and 10 µg LHD153). Groups of 8 New Zealand White rabbits (female, at least 2.5 kg in weight) were vaccinated intramuscularly with 15 µg of GMMA (OAg dose based on HPAEC-PAD quantification) formulated either in saline or Alhydrogel with a volume of 500 µL on study days 0 and 28. Sera were collected on days 1, 27, and 42 (individual serum samples). Body weight and temperature were monitored following vaccination for 72 h (body weight) or 24 h (temperature) or until the original values were restored in case body weight loss was >10% and temperature >40 °C.

Animal health conditions were monitored throughout all the studies, and no clinical signs of concern were reported in mice and rabbits.

### 2.5. Ethics and 3R Statement

All animal experiments were performed in accordance with relevant national and international legislation (Italian Legislative Decree 26/2014 and European Directive for the Use of Animals for Scientific Purposes 2010/63) and GSK Animal Welfare Policy and standards. All animal protocols were reviewed by the local Animal Welfare Body and approved by the Ministry of Health, according to the abovementioned legislation.

GSK is committed to the replacement, reduction, and refinement of animal studies (3Rs). Nonanimal models and alternative technologies are part of our strategy and are employed where possible. When animals are required, the application of robust study design principles and peer review minimises animal use, reduces harm, and improves the benefit in studies.

### 2.6. Assessment of Anti-S. sonnei LPS and Anti-GMMA Protein Immune Responses in Mice and Rabbits

Preimmune sera and sera collected four weeks after the first immunisation and two weeks after the second immunisation were analysed by ELISA [30] to determine anti-*S. sonnei* LPS total IgG, IgM, and IgG subclasses’ content using *S. sonnei* LPS as the plate-coating antigen (at the concentration of 0.5 µg/mL in phosphate buffer saline (PBS)) and anti-*Shigella* GMMA proteins’ total IgG, IgM, and IgG subclasses’ content using the *S. sonnei* OAg-negative GMMA as the plate-coating antigen (at the concentration of 15 µg/mL in PBS). The results are expressed in ELISA units determined relative to the standard serum. One ELISA unit (EU) equals the reciprocal of the dilution of the standard serum giving an OD_405–490nm_ of 1 in the standard assay.

### 2.7. Assessment of Serum Bactericidal Activity against Shigella

Individual serum samples collected on day 27 and/or day 42 were assayed through a luminescent-based serum bactericidal assay (SBA), as previously described against *S. sonnei* [30]. Heat-inactivated (HI) sera were serially diluted in PBS in the SBA plate (25 µL/well). The starting dilution of each serum in the assay was 1:100 (final dilution), followed by three-fold dilution steps up to seven dilution points, plus one control well with no sera added. A 4-parameter nonlinear regression was applied to the raw luminescence obtained (no normalisation of data was applied) for all serum dilutions tested for each serum; an arbitrary serum dilution of 10^15^ was assigned to the well containing no sera. Fitting was performed by weighting the data for the inverse of luminescence^2 and using GraphPad Prism 7 software (GraphPad Software, Boston, MA, USA).

The results of the assay are expressed as IC50 values, represented by the reciprocal serum dilution that is able to reduce the luminescence signal by 50% compared to the negative control (and thus causes 50% growth inhibition). Titres lower than the minimum measurable assayed dilution were assigned a value of half of the first dilution of sera tested (50).

### 2.8. Statistical Analysis

Dose-dependent responses in mice were evaluated for (a) *S. sonnei* LPS-specific total IgG, (b) *S. sonnei* GMMA protein-specific total IgG, and (c) *S. sonnei* bactericidal titres. For the curve comparison using the parallel line approach with the possibility of changing the dose range (PLAS), the data obtained were log-transformed to represent each curve with log-transformed doses on the abscissa and the log-transformed ELISA units, or SBA titres, on the ordinate (natural log was used for both transformations). For each formulation type, three out of four consecutive doses were used by selecting the dose range that provided a higher slope for the linear curve; no weight function was used for the regression. For each formulation type, the chosen dose range in the comparison could be different (PLAS). The nonparallelism and nonlinearity of the lines were checked for the different formulations. If nonparallelism or nonlinearity was verified, no comparison was performed. Otherwise, the relative potency of the two compared formulations was calculated with a 95% confidence interval. CombiStats version 7.0 and GraphPad version 7.00 were used.

## 3. Results

### 3.1. GMMA Formulations

*S. sonnei* GMMA, produced and purified as previously reported [13], were characterised by an OAg-to-protein weight ratio of 0.29 and had a Z average particle diameter of 160.4 nm based on dynamic light scattering, with a polydispersity index of 0.16. Soluble proteins represented 5.7% of the total protein, as determined by microBCA, and the lipid A content on the total OAg was 0.5 nmol/μg (by HPLC-RP MS/HPAEC-PAD) [28]. OAg displayed on GMMA was characterised by populations at different molecular weights: group 4 capsule and high-molecular-weight OAg at 234 kDa, a population of medium-molecular-weight OAg at 19.2 kDa, and a low-molecular-weight OAg population around 2.2 kDa.

When GMMA were formulated with Alhydrogel, analysis via the silver staining of the supernatant confirmed the total adsorption of GMMA. The same factor was verified with AS37, where, additionally, <1% LHD153 was found to be not adsorbed on Alhydrogel by HPLC-RP analysis. The LHD153 dose selected for the animal studies presented in this manuscript was determined based on previous work by Buonsanti et al. [29].

### 3.2. Comparing GMMA Formulations in Mice

**Figure 1:**
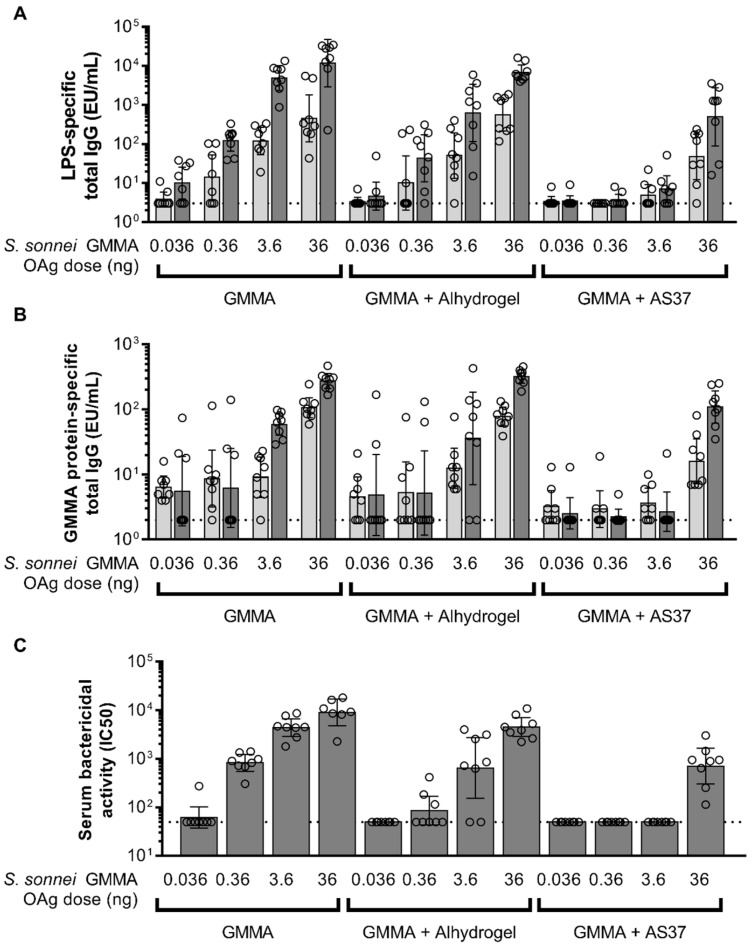
*S. sonnei*-specific humoral responses elicited upon vaccination with GMMA either alone or with Alhydrogel or AS37. Five-week-old CD1 mice were immunised intramuscularly (50 µL, 25 µL/leg) on study days 0 and 28. Mice were bled on study days −1, 27, and 42. LPS-specific (**A**) and *S. sonnei* GMMA protein-specific (**B**) total IgG responses were quantified by ELISA. Antibody complement-mediated functionality in killing *S. sonnei* (**C**) was assessed by SBA. The GMMA dose (quantified as total OAg) received by animals in each immunisation group is reported below each graph. Individual IgG levels are reported in the graphs (symbols) together with geometric means (bars) and 95% CI. Light grey bars represent the geometric means after the first immunisation, whereas dark grey bars represent the geometric means after the second immunisation. Dotted lines represent the level of antibodies (**A**,**B**) or the bactericidal activity (**C**) of preimmune sera.

The different GMMA formulations were compared in mice at four different OAg doses in the range of 0.036–36 ng. The doses were selected based on previous results to have a dose–response in the range chosen. LPS-specific and GMMA protein-specific total IgG elicited using intramuscular vaccination were quantified through ELISA, while serum functionality was assessed against the *S. sonnei* wild-type strain through SBA (Figure 1).

Unadsorbed and Alhydrogel-adsorbed *S. sonnei* GMMA elicited significantly higher LPS-specific total IgG levels than the baseline already 27 days after the first vaccination at all the tested doses but the lowest one. AS37-adsorbed *S. sonnei* GMMA elicited LPS-specific total IgG levels that were above the preimmune serum baseline only at the two highest tested doses after one vaccination. After the second vaccination, all groups revealed an anti-LPS IgG response above the preimmune serum baseline (Figure 1A). Similar results were obtained for anti-GMMA protein total IgG (Figure 1B).

After the second immunisation, antibodies elicited by immunisation with the three highest doses of unadsorbed GMMA and the two highest doses of Alhydrogel-adsorbed GMMA mediated the killing of the *S. sonnei* strain in SBA, whereas only antibodies elicited after immunisation with the highest dose of AS37-adsorbed GMMA showed functional titres (Figure 1C).

**Figure 2:**
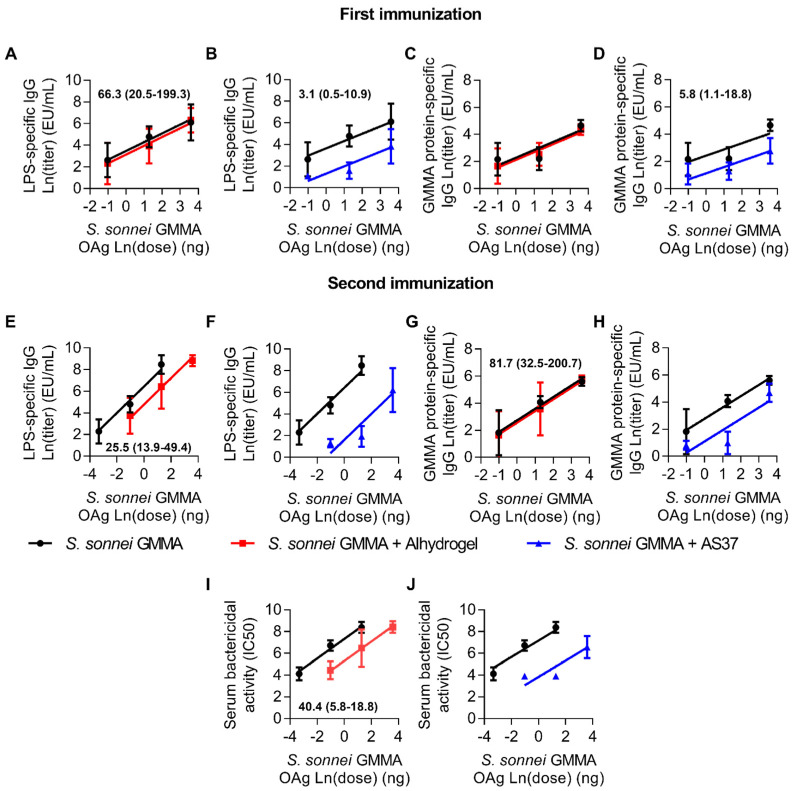
Comparison of *S. sonnei*-specific humoral responses elicited upon vaccination with GMMA either in the absence or presence of the two tested adjuvants. Five-week-old CD1 mice were immunised intramuscularly (50 µL, 25 µL/leg) on study days 0 and 28. Mice were bled on study days-1, 27, and 42. LPS-specific (**A**,**B**,**E**,**F**) and *S. sonnei* GMMA protein-specific (**C**,**D**,**G**,**H**) total IgG responses and antibody complement-mediated functionality in killing *S. sonnei* (**I**,**J**) observed in mice immunised with unadsorbed *S. sonnei* GMMA (black lines) were compared to those elicited by *S. sonnei* GMMA adsorbed on Alhydrogel (red lines) or *S. sonnei* GMMA adsorbed on AS37 (blue lines) by the parallel line approach with the possibility of changing the dose range (PLAS). Each curve is represented with the log-transformed dose on the abscissa and the log-transformed ELISA units or SBA titres on the ordinate (natural log was used for both transformations). For each formulation type, three out of four consecutive doses were used by selecting the dose range that provided a higher slope for the linear curve; no weight function was used for the regression. For each formulation type, the chosen dose range in the comparison could be different (PLAS). The calculated relative potency with a 95% confidence interval is reported in the graph when applicable. Relative potency was not calculated when the dose–response was evaluated as nonlinear.

In the entire range of doses tested, unadsorbed GMMA elicited an LPS-specific total IgG response of greater magnitude than GMMA adsorbed on Alhydrogel after the second immunisation, which translated to higher bactericidal titres as well (Figure 2A,C,E,G,I). By contrast, no differences were detected for the LPS-specific total IgG response after the first immunisation and for GMMA protein-specific responses at all the tested time points. In addition, unadsorbed GMMA elicited higher humoral responses (LPS-specific, GMMA protein-specific, and functionality) than GMMA formulated with AS37 at all the tested time points (Figure 2B,D,F,H,J). Overall, the addition of Alhydrogel or AS37 impaired the antigen-specific humoral response elicited by vaccination with *S. sonnei* GMMA.

To fully characterize the immune response elicited by the different formulations, sera derived from immunisation with the highest dose (36 ng OAg) of GMMA were also analysed for IgM and IgG subclasses.

**Figure 3:**
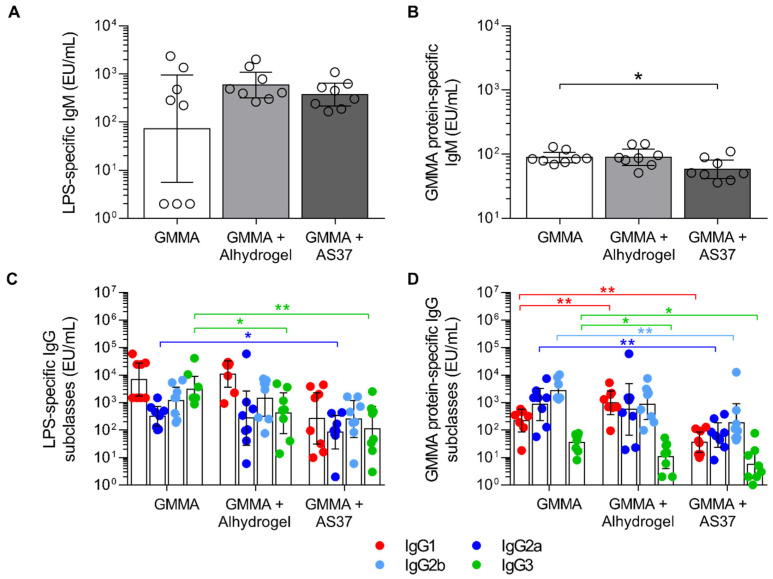
*S. sonnei*-specific IgG subclasses and IgM elicited upon two vaccinations with GMMA either alone or adsorbed on Alhydrogel or AS37. Five-week-old CD1 mice were immunised intramuscularly (50 µL, 25 µL/leg) on study days 0 and 28 with 36 ng of *S. sonnei* GMMA (quantified as OAg). Mice were bled on study days −1, 27, and 42. LPS-specific (**A**–**C**) and *S. sonnei* GMMA protein-specific (**B**–**D**) IgM and IgG subclasses were quantified by ELISA. Geometric means of each IgG subclass level are reported in graphs (**A**,**B**) (bars), whereas individual IgM levels are reported in graphs (**C**,**D**) (symbols) together with geometric means (bars) and 95% CI. * *p* < 0.05, ** *p* < 0.01; Mann–Whitney test.

The levels of LPS-specific IgM were comparable in the sera of mice of the three immunisation groups, even if some animals showed no detectable IgM after immunisation with unadsorbed GMMA. The levels of GMMA protein-specific IgM were lower in mice immunised with *S. sonnei* GMMA+AS37 in comparison to unadsorbed GMMA (Figure 3A,B).

Looking at IgG subclasses (Figure 3C,D), unadsorbed *S. sonnei* GMMA induced significantly higher levels of LPS-specific IgG3 than Alhydrogel-adsorbed GMMA, whereas the levels of the other IgG subclasses were comparable. Also, unadsorbed GMMA induced significantly higher levels of LPS-specific IgG3 and IgG2a than GMMA adsorbed on AS37, while levels of IgG1 and IgG2b were comparable. GMMA protein-specific IgG1 levels were higher in mice that were immunised with GMMA adsorbed on Alhydrogel than in mice injected with unadsorbed GMMA, whereas the opposite occurred in GMMA-protein-specific IgG3. AS37-adsorbed GMMA elicited lower levels of GMMA protein-specific subclasses in comparison to unadsorbed GMMA.

By comparing the bactericidal activity of sera raised against the three formulations at the highest tested dose, we found that functional titres were higher in the group of mice immunised with unadsorbed GMMA compared to GMMA adsorbed on Alhydrogel and AS37 (*p* < 0.05 and *p* < 0.001, respectively). Interestingly, the overall analysis of the results obtained showed that SBA titres correlated with the total IgG response but not with IgM; IgG1 and IgG3 better correlated with the ability to kill the bacterium (Table 1).

### 3.3. Comparing GMMA Formulations in Rabbits

**Figure 4:**
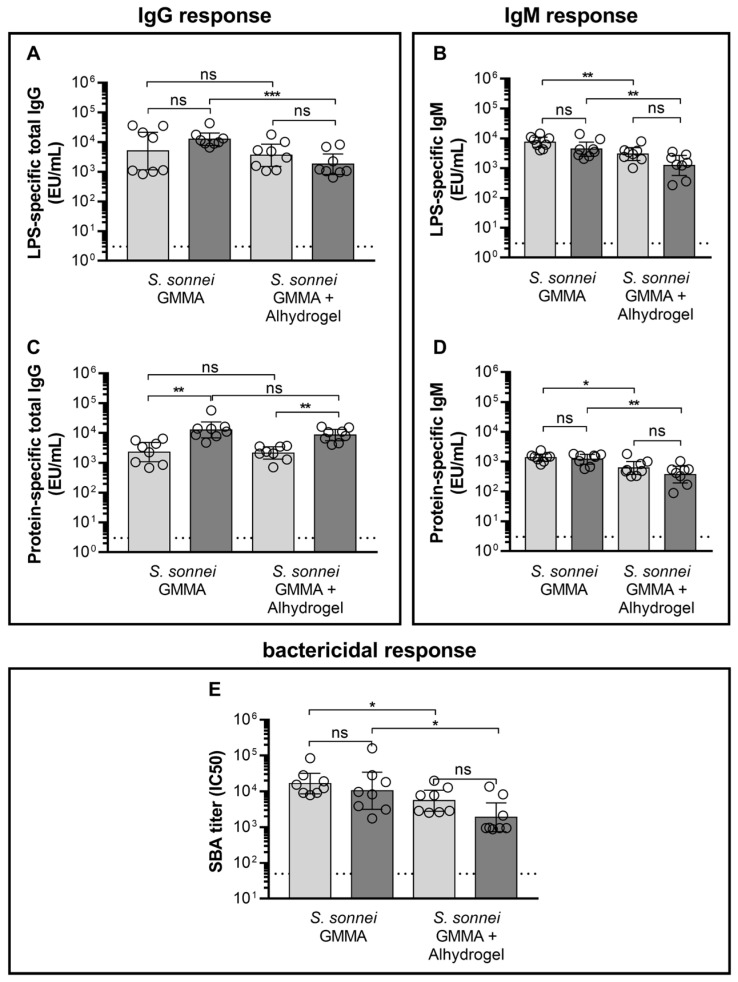
*S. sonnei*-specific total IgG and IgM levels elicited upon two vaccinations with *S. sonnei* GMMA either unadsorbed or adsorbed on Alhydrogel. Female New Zealand White rabbits were vaccinated intramuscularly (0.5 mL) on study days 0 and 28 with 15 µg of *S. sonnei* GMMA (quantified as total OAg). LPS-specific (**A**,**B**) and *S. sonnei* GMMA protein-specific (**C**,**D**) total IgG and IgM were quantified by ELISA. Antibody complement-mediated functionality in killing *S. sonnei* (**E**) was assessed by SBA. Individual IgG and IgM levels are reported together with geometric means (bars) and 95% CI. * *p* < 0.05, ** *p* < 0.01, *** *p* < 0.001; Mann–Whitney test. A paired *t*-test was performed between different time points, and an unpaired *t*-test was performed between different immunisation groups. Light grey bars represent the geometric means after the first immunisation, whereas dark grey bars represent the geometric means after the second immunisation. Dotted lines represent the level of antibodies (**A**–**D**) or the bactericidal activity (**E**) of preimmune sera.

Based on the results in mice, unadsorbed GMMA and GMMA adsorbed on Alhydrogel were selected to be compared in rabbits. Female New Zealand White rabbits were vaccinated intramuscularly with *S. sonnei* GMMA at 15 µg of OAg, which is the dose of *S. sonnei* GMMA component contained in the four-component GMMA-based vaccine against *Shigella* currently tested in a phase 1/2 trial (clinicaltrial.gov NCT05073003). LPS-specific and GMMA protein-specific total IgG and IgM elicited by vaccination were quantified through ELISA, while serum functionality against the *S. sonnei* wild-type strain was determined through SBA (Figure 4).

LPS-specific and GMMA protein-specific total IgG levels elicited after the first immunisation by GMMA adsorbed on Alhydrogel were comparable to those elicited by unadsorbed GMMA. LPS-specific total IgG levels elicited after the second injection with GMMA adsorbed on Alhydrogel were instead significantly lower than those elicited by unadsorbed GMMA, whereas GMMA protein-specific total IgG levels remained comparable (Figure 4A,C).

LPS-specific and GMMA protein-specific IgM levels elicited both after the first and second immunisation by GMMA adsorbed on Alhydrogel were lower than those elicited by unadsorbed GMMA (Figure 4B,D).

Finally, the antibody functionality paralleled the results obtained for LPS-specific IgG and IgM (Figure 4E), with unadsorbed GMMA eliciting higher SBA titres than GMMA adsorbed on Alhydrogel.

**Figure 5:**
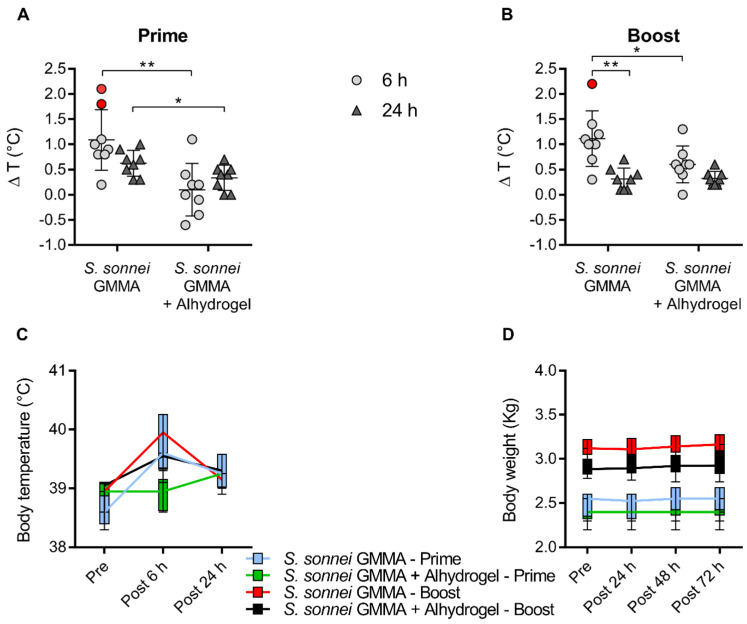
Rabbit body temperature and weight changes after vaccination with *S. sonnei* GMMA either unadsorbed or adsorbed on Alhydrogel. Female New Zealand White rabbits were vaccinated intramuscularly (0.5 mL) on study days 0 and 28 with 15 µg of *S. sonnei* GMMA (quantified as OAg). Rabbit body temperature (**A**–**C**) was monitored before and 6 and 24 h after each immunisation. Body weight (**D**) was measured before immunisation and 24, 36, and 72 h after each vaccination. Mean and standard deviation for all groups and individual values of ∆T (°C) are reported in graphs (**A**,**B**) (∆T > 1.5 in red). Changes in T (°C) and weight (Kg) during the time after vaccination are reported in box and whisker graphs (plotting min to max values, (**C**,**D**)). * *p* < 0.05, ** *p* < 0.01; A paired parametric *t*-test was performed between different time points, and an unpaired parametric *t*-test was performed between different immunisation groups.

Rabbits injected with either *S. sonnei* GMMA adsorbed on Alhydrogel or *S. sonnei* GMMA alone showed a limited and transient body temperature increase, returning close to baseline values within 24 h after each vaccination. Indeed, the average difference in temperature 24 h post-vaccination with *S. sonnei* GMMA adsorbed on Alhydrogel compared to baseline values was 0.3 °C, while the average difference in temperature 24 h post-vaccination with *S. sonnei* GMMA alone compared to baseline values was 0.6 °C after the first immunisation and 0.3 °C after the second immunisation. The maximum body temperature increase was 1.3 °C and 2.2 °C at 6 h post-injection for *S. sonnei* GMMA adsorbed on Alhydrogel and *S. sonnei* GMMA alone, respectively. We observed a significantly reduced temperature rise 6 h after immunisation with *S. sonnei* GMMA adsorbed on Alhydrogel compared to *S. sonnei* GMMA alone (Figure 5A,B). However, the difference in terms of absolute temperature values between Alhydrogel-adsorbed and unadsorbed GMMA was low (Figure 5C), assuming that 38–40 °C represents the normal body temperature range for New Zealand White rabbits. No body weight loss was observed in any rabbit for any of the immunisation groups following each vaccination and at study termination, confirming the good clinical conditions of the study animals (Figure 5D). Overall, immunisation with unadsorbed GMMA did not raise any concern for animal health.

## 4. Discussion

Alhydrogel has been used in preclinical and clinical studies with *S. sonnei* GMMA vaccine formulation as an adsorbent agent with the main purpose of reducing potential GMMA systemic reactogenicity. In this study, we performed an in-depth investigation on the impact of aluminium salt-based adjuvants such as Alhydrogel or AS37 on *S. sonnei* GMMA immunogenicity and evaluated for the first time the tolerability of GMMA formulated without Alhydrogel in rabbits. In the last few years, several synthetic TLR agonists have been selected as molecules that can be used as vaccine adjuvants and have been tested in clinical trials. One example is the TLR4 agonist monophosphoryl lipid A (MPL), a detoxified form of bacterial LPS. MPL formulated with aluminium hydroxide, named AS04, has been approved for use in vaccines that target human papillomavirus and hepatitis B [31]. Other TLR agonists that have demonstrated promising preclinical and clinical results as vaccine adjuvants are those targeting the TLR7/8 pathway [29,32]. However, GMMA possess the ability to stimulate the innate compartment of a host’s immune system through the activation of TLRs by various bacterial PAMPs such as LPS, lipoproteins, flagellin monomers, and bacterial DNA fragments [14]. Indeed, in recent studies, the intramuscular injection of OMVs with heterologous antigens enhanced antigen-specific humoral and cellular immune responses and increased the protection rate against tumour and virus challenges [33,34]. Therefore, we only tested AS37 as an adjuvant together with Alhydrogel since it contains a TLR agonist that should not be present on GMMA and can eventually synergise with those on GMMA and still has an absorbent function being an aluminium salt-based adjuvant.

In the two animal species tested in this study, the immunogenicity of unadsorbed *S. sonnei* GMMA was found to be satisfactory, and the addition of aluminium salt-based adjuvants did not improve the magnitude, quality, or functionality of vaccine-elicited antibodies. On the contrary, the addition of adjuvant impaired GMMA immunogenicity. This effect may be due to the overstimulation of the immune system when the adjuvant is added to GMMA or a different mechanism of action of GMMA when adsorbed on aluminium salt-based adjuvants compared to unadsorbed GMMA. For example, we demonstrated that antigen presentation by follicular dendritic cells (FDCs) to cognate B cells plays a central role in *Shigella* GMMA immunogenicity [22]. Thus, we can hypothesise that entrapping GMMA vesicles at the injection site, by using aluminium salt formulations, might be detrimental to GMMA immunogenicity because of reducing the translocation of GMMA vesicles to draining lymph nodes, where they are captured by FDCs. Moreover, we showed that the presence of aluminium salt-based adjuvants reduced the levels of IgG3 induced by GMMA in mice, which had a strong correlation with bactericidal activity (Table 1), and this may be a possible explanation for the observed results. Consistently, we have previously demonstrated in a Meningococcus B model (MenB) that, when immunising mice with GMMA harbouring the antigen factor H-binding protein (fHbp) on the surface, the bactericidal activity mediated by fHbp was increased compared to fHbp alone or fHbp physically mixed with GMMA, and this increase was associated with the higher production of IgG subclasses with superior bactericidal potential against MenB, including IgG3 [35].

We have previously shown a correlation between SBA titres and anti-*S. sonnei* LPS serum IgG levels in subjects vaccinated with a single-component candidate vaccine against *S. sonnei* (1790GAHB) [36]. In this work, we have shown for the first time that the same correlation between LPS-specific serum IgG and bactericidal activity is also observed in mice and that, in this animal species, bactericidal activity mainly correlates with IgG1 and IgG3 subclasses. We did not evaluate the correlation of GMMA protein-specific IgG with bactericidal activity since we know from previous studies that anti-OAg IgG is the main driver of bactericidal activity against OAg-positive *S. sonnei* bacteria [30,36].

Alhydrogel has been used with GMMA mainly to reduce their potential reactogenicity. The reactogenicity of OMV-based vaccines has been found to be modulated by adsorption to aluminium salt when assessed in rabbit pyrogenicity tests (RPTs). Indeed, Kaaijk et al. compared plain and aluminium-adsorbed MenB OMVs (NonaMen) in a modified RPT, detecting a transient temperature rise of approximately 0.6 °C 4 h after the first immunisation with both MenB vaccines and a 0.2–0.3 °C rise in body temperature at the same time point upon second vaccination just with plain NonaMen, with no increase in body temperature being detected with the aluminium-adsorbed NonaMen vaccine [37].

The preliminary safety data in rabbits collected in this work showed that unadsorbed GMMA were well tolerated and caused only a mild and transient temperature rise after the first and second vaccinations. The use of animals of the same sex (females) to assess GMMA immunogenicity and tolerability, and thus not taking into account sex as a parameter, might have influenced the results we obtained; we acknowledge that this may be considered a study limitation. A pivotal future GLP toxicity study will be conducted in rabbits of both sexes, allowing for a thorough assessment of both safety and immunogenicity parameters.

In addition, as the assessment of the temperature increase in rabbits is considered a pillar test to evaluate the systemic reactogenicity in toxicology studies, our data strongly suggest that it would be useful to test GMMA with no Alhydrogel in healthy adults in order to verify whether the absence of aluminium salt formulation may not be a safety concern by inducing better immunogenicity of GMMA-based vaccines at the same time. Indications that GMMA that are not adsorbed on Alhydrogel may have a good safety profile in humans are also inferred from a monocyte activation test (MAT), developed as an alternative to RPTs for monitoring the pyrogenicity of *Shigella* GMMA-based vaccines, which showed that GMMA not adsorbed on Alhydrogel induce modest levels of IL-6 release by human monocytes [38]. Indeed, although it is still hard to determine the gold standard preclinical model to fully predict reactogenicity in humans, the MAT has the clear advantage of using human cells and therefore allows for the measurement of the activation of human TLRs [39]. Moreover, the vaccine based on native OMVs prepared from a genetically detoxified mutant of group B *Neisseria meningitidis* was tested in humans and was found to be well tolerated. Injection site reactions were not dose-related and appeared to be worse in those receiving aluminium hydroxide. In addition, the incidence of seroconversion was not significantly increased by adding the aluminium hydroxide adjuvant [40].

The removal of Alhydrogel from GMMA-based vaccine formulation will simplify vaccine manufacturing and further reduce costs. Moreover, the absence of Alhydrogel could facilitate the formulation process of the combination of GMMA derived from different pathogens in order to develop more complex combination vaccines. Our study suggests that the absence of aluminium salts in GMMA-based vaccines is not detrimental to the safety and immunogenicity of such vaccines and supports the comparison of GMMA-based vaccines formulated in the presence or absence of aluminium salts in clinical trials.

## Figures and Tables

**Table 1:** Correlations among *S. sonnei* LPS-specific IgG/IgM responses and bactericidal activity. Five-week-old CD1 mice were immunised intramuscularly (50 µL, 25 µL/leg) on study days 0 and 28 with 36 ng of *S. sonnei* GMMA (quantified as total OAg) either alone or adsorbed on Alhydrogel or AS37 and bled on study day 42. The *S. sonnei* LPS-specific IgG response detected in all mice was correlated with serum bactericidal activity against *S. sonnei*. Pearson correlation coefficients (and *p*-values of the correlation) are reported for total IgG and IgM and each IgG subclass.

	Pearson Correlation Coefficients vs. Bactericidal Activity (and *p*-Values of the Correlation)
**IgG**	0.923 (0.000)
**IgM**	−0.363 (0.089)
**IgG2a**	0.532 (0.009)
**IgG1**	0.629 (0.001)
**IgG2b**	0.505 (0.014)
**IgG3**	0.714 (0.000)

## Data Availability

The original contributions presented in the study are included in the article; further inquiries can be directed to the corresponding author/s.
